# When to think of genetic causes for rhabdomyolysis? A Brazilian single-center exploratory study

**DOI:** 10.1055/s-0046-1827166

**Published:** 2026-08-13

**Authors:** Aldrin Pedroza Martins, Alberto Rolim Muro Martinez, Anamarli Nucci, Marcondes Cavalcante França Júnior

**Affiliations:** 1 Universidade Estadual de Campinas, Faculdade de Ciências Médicas, Departamento de Neurologia, Campinas SP, Brazil.

**Keywords:** Rhabdomyolysis, Muscular Dystrophies, Neuromuscular Diseases, Myalgia, Muscular Diseases

## Abstract

**Background:**

Rhabdomyolysis is a potentially life-threatening condition that can result from genetic causes. Despite that, little is known about the genetic underpinnings behind monogenic rhabdomyolysis in Brazil.

**Objective:**

To address the frequency and predictive factors for monogenic causes of rhabdomyolysis in a Brazilian series.

**Methods:**

Patients presenting with rhabdomyolysis at Universidade Estadual de Campinas (UNICAMP) from April 2024 to October 2025 were selected. Rhabdomyolysis was defined when (1) there was at least 1 episode of creatine kinase (CK) levels ≥ 5x upper limit of normal (ULN) not better explained by exogeneous factors and (2) at least 1 of the RHABDO criteria (R - recurrent episodes of exertional rhabdomyolysis; H - hyperCKemia persisting more than 8 weeks after event; A: accustomed physical exercise; B - blood creatine kinase (CK) > 50x ULN; D – drug ingestion/medication/supplements or other exogenous and endogenous factors cannot sufficiently explain the rhabdomyolysis severity; O - other family members affected / other exertional symptoms [e.g., cramps or myalgia]). was met. A 2-step genetic investigation was undertaken: first a 50-gene panel enriched in metabolic myopathy genes and for those with negative results, whole exome sequencing (WES). Clinical and demographic data of patients with and without gene etiology were then compared.

**Results:**

We identified 20 patients, 8 of whom were men, with mean age at the first episode of rhabdomyolysis of 19.5 (2–50) years old. Monogenic etiology was found in 9 of the patients. The associated genes were *PYGM* (most frequent), *CPT II* , *ACADS* , *ACADM* , *ANO5* , *DYSF* and *FKTN* . Creatine kinase ≥ 5x ULN persisting for more than 8 weeks after the event ( *p* = 0.0081), myalgia ( *p* 0.028), objective weakness ( *p* = 0.049) and parental consanguinity ( *p* = 0.0090) were more frequent in the group with genetic cause.

**Conclusion:**

After properly exclusion of exogenous etiologies, monogenic causes accounted for nearly half of the cases of rhabdomyolysis. Genetic testing should be pursued in those patients with persistent CK elevation, presence of myalgia, objective weakness or parental consanguinity.

## INTRODUCTION

Rhabdomyolysis is a potentially life-threatening medical condition characterized by skeletal muscle cell damage leading to the release of internal components into the bloodstream. 1 Following muscle cell membrane injury, the serum levels of creatine kinase (CK), myoglobin, and stored ions (mainly potassium) increases. The accumulation of these components into the blood, particularly myoglobin, is responsible for acute kidney injury, the major cause of morbidity related to rhabdomyolysis. 2

The serum level of CK is a useful biomarker to assess severity and response to treatment. Although not a worldwide consensus, CK serum level is often part of the definition of rhabdomyolysis. Two previous reports of large cohorts determined the minimum cutoff of at least 5x upper limit of normal, 3 4 recently confirmed by a systematic review. 5 Often, the increase of CK is accompanied with myalgia, exercise intolerance, cramps, muscle swelling and myoglobinuria (dark urine). 6

The set of possible causes for the syndrome of rhabdomyolysis is vast and can be divided into two groups: acquired (trauma, drug abuse, medication, poisoning, fever/infectious, extreme physical exertion) and genetic (inherited myopathies, such as metabolic, mitochondrial, muscular dystrophies, and channelopathies). 7 8 In the past 10 years, the comprehension of the genetic landscape has expanded, with a growing body of evidence supporting the concept of genetic susceptibility behind many rhabdomyolysis cases. 9 10 11 In this context, some international efforts worldwide were launched to map the most common genes involved, the phenotypic profiles of specific genes and the predictive factors related to a higher risk of monogenic causes. 12 13 14 Hence, based on expert opinion, some criteria have been proposed to identify genetic propensity/cause for rhabdomyolysis. 15 16 In 2016, Scalco et. al 16 introduced the acronym RHABDO (R - recurrent episodes of exertional rhabdomyolysis; H - hyperCKemia persisting more than 8 weeks after event; A: accustomed physical exercise; B - blood creatine kinase (CK) > 50x ULN; D – drug ingestion/medication/supplements or other exogenous and endogenous factors cannot sufficiently explain the rhabdomyolysis severity; O - other family members affected / other exertional symptoms [e.g., cramps or myalgia]) to highlight predictors of an underlying genetic cause.

Much has been learned about monogenic rhabdomyolysis, but surprisingly little is known about the genetic landscape in Brazilian cohorts. So far, no local cohort with a comprehensive genetic investigation was reported. It remains unclear whether the RHABDO criteria are indeed useful in our population. Hence, the current study was designed to tackle these open questions. We recruited a well-characterized cohort from a national neuromuscular reference center who underwent a comprehensive genetic investigation. Our ultimate goals were to identify the frequency as well as predictive factors for monogenic causes of rhabdomyolysis in this Brazilian series.

## METHODS

### Subjects' selection and clinical data collection

**Fig. 1:**
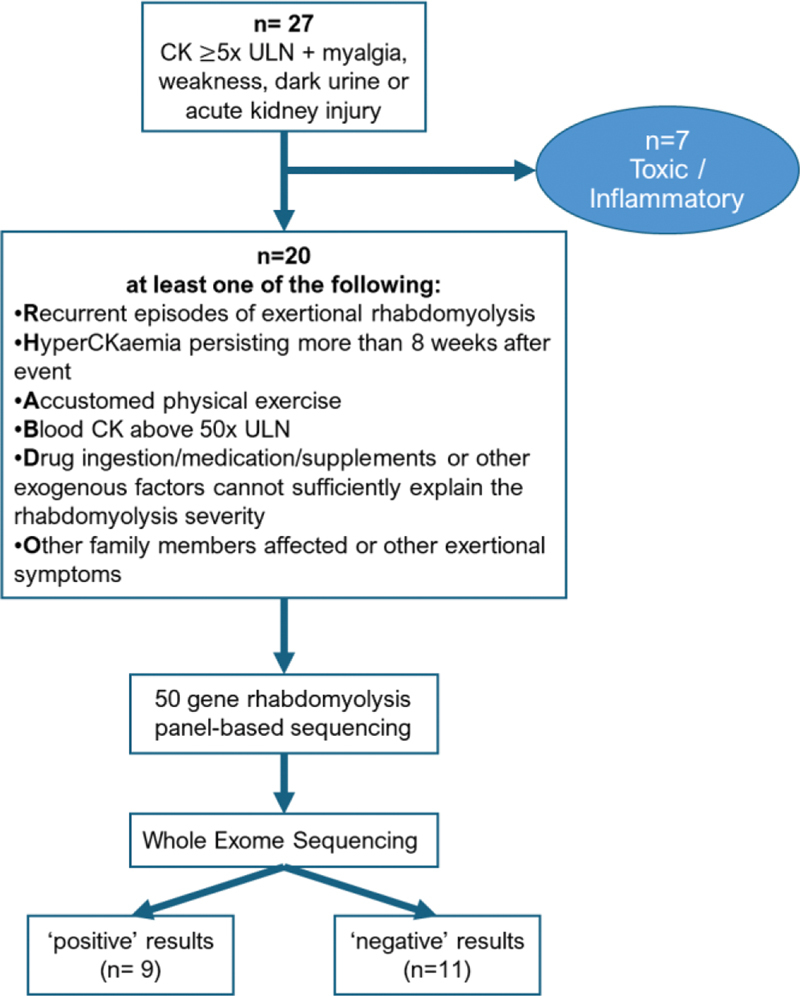
Abbreviations: ULN, upper limit of normal; CK, creatine kinase; WES, whole exome sequencing. Flowchart showing the inclusion and exclusion criteria and sequential genetic testing performed along with their diagnostic yield.

All patients who visited the neuromuscular clinic at Universidade Estadual de Campinas (UNICAMP) between April 2024 to October 2025 were screened for any previous or ongoing report of serum CK level above 5x upper limit of normal (ULN) associated to at least 1 of the following: myalgia, weakness complaint, dark urine, or acute kidney injury. Whenever inflammatory or toxic causes were identified, patients were excluded from further analyses. Then, from the remaining patients, we selected those meeting at least one of the RHABDO criteria. This group underwent detailed genetic investigation (described below). The study flowchart is depicted in Figure 1 .

For each patient, demographic as well as clinical information was collected including sex, current age, age at first episode of rhabdomyolysis, CK peak at first episode, CK peak at least 8 weeks after the event, serum CK level > 5x ULN at least 8 weeks after the event, recurrence of rhabdomyolysis, presence of exogeneous trigger, fatigue, exercise intolerance, myalgia, cramps, objective weakness, family history (e.g., cramps, myalgia, exercise intolerance, diagnosis of genetic myopathy), and the presence of comorbidities (e.g., epilepsy, cognitive impairment, heart failure, ventilatory impairment, endocrinopathy).

The current study was approved by the Ethics Committee from the Clinic Hospital of UNICAMP, and an informed consent was obtained from the patients. The study was performed in compliance with the principles of the Declaration of Helsinki.

### Genetic testing

We collected either blood samples or buccal smears from these patients. Afterwards, genomic deoxyribonucleic acid (DNA) was extracted. A 2-step genetic investigation was then undertaken: first a 50-gene panel enriched in metabolic myopathy genes and for those with negative results, WES. All genes included in the panel are available in Supplementary Material Box 1 (available at https://www.arquivosdeneuropsiquiatria.org/wp-content/uploads/2026/05/ANP-2026.0012-Supplementary-Material.docx ). Variants classified as of undetermined significance (VUS), pathogenic (P), or likely pathogenic (LP), according to the American College of Medical Genetics and Genomics (ACGM) criteria, were retrieved.

Patients were then divided into two groups based on panel/WES results: Those with positive and those with negative results. Demographic and clinical variables were then compared in the two groups in an attempt to identify predictors of an underlying genetic etiology for rhabdomyolysis.

### Statistical analyses

Data were presented using descriptive statistics. Categorical data were compared using Fisher's exact test, whereas continuous variables were compared using the Mann-Whitney U test. The level of significance was set at *p* < 0.05. All analyses were performed on GraphPad Prism version 10.4.2 for Mac (GraphPad Software, Inc.).

## RESULTS

### Clinical and genetic findings

From April 2024 to October 2025, we identified 27 patients with a history of rhabdomyolysis. However, seven were excluded because there was an alternative cause for increased CK (three cases of toxic myopathy and four of inflammatory myopathy). The three cases deemed as toxic had subacute mild weakness onset related to statins exposure with good prognosis after drug discontinuation. Among the 4 inflammatory cases, there were also 2 statin-induced (2 immune-mediated necrotizing myopathies, with 1 being positive for anti-signal recognition particle [anti-SRP], and the other positive for anti-3-hydroxy-3- methylglutaryl coenzyme A [anti-HMG-CoA] reductase antibody). The other two had dermatomyositis features (one was anti-TIF1-γ positive and the other negative for antibody screening).

Then, we were left with 20 subjects for genetic investigation ( Figure 1 ). There were 12 women and 8 men. The median age at the 1st episode of rhabdomyolysis and at the 1st evaluation at UNICAMP were 19.5 (2–50) and 26.5 (6–60) years, respectively. Twelve patients (60%) had rhabdomyolysis as the heralding manifestation, whereas the remaining 8 met the criteria employed in this analysis during their follow-up. Fifteen patients (75%) reported some external trigger as the major cause of rhabdomyolysis, with exercise being the most common (73.3%) followed by infection/fever (26.7%). Rhabdomyolysis was recurrent in 16 (80%) patients.

**Fig. 2:**
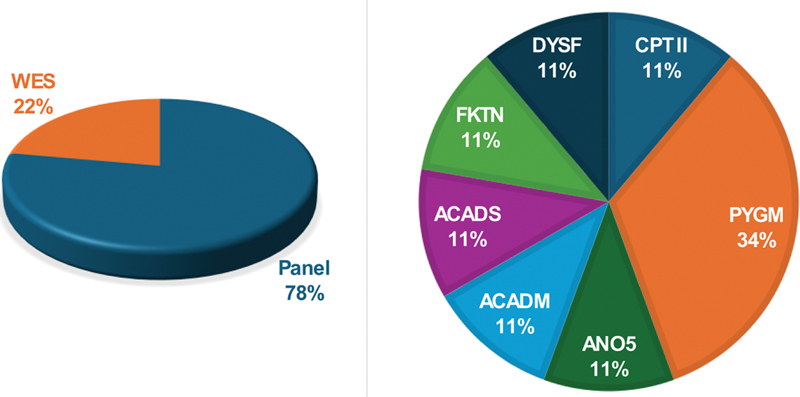
Proportion of diagnosis achieved by genetic test strategy (A) and relative frequency of genes found to be causative for underlying myopathy (B).

Genetic diagnosis was reached in 9 out of the 20 patients (45%), 7 of them using the gene panel and 2 using WES. We recovered 20 variants in 17 different genes. Out of them, 7 variants were classified as P or LP (7 patients) and 13 as VUS (6 patients). Among the last ones, three were deemed as contributing to the phenotype and, therefore, two patients were included in the group with positive genetic testing. These patients and their variants are indicated in Table 1 . The same pathogenic variant in the *PYGM* gene was identified in three different patients, and it was the most common one in the sample. Altogether, 7 genes and 10 different variants were identified as causative of an underlying myopathy related to the rhabdomyolysis event. The genotype details are shown in Table 2 , whereas the proportion of the genetic test performed, and the relative frequency of the genes found are represented in Figure 2 .

**Table 1:** Variant description and classification, genetic test performed and summary of RHABDO features of patients who had some candidate variant found

Patient ID	Gene	RefSeq (NM_)	Variant (cDNA)	Zygosity	ACGM classification	Genetic test	RHABDO criteria
1	*CPT II*	000098.3	c.110_111dup	Heterozygous	5	Panel	RABD
		000098.3	c.338C > T	Heterozygous	5		
2	*PYGM*	005609.4	c.148C > T	Homozygous	5	WES	RHABDO
3	*PYGM*	005609.4	c.148C > T	Homozygous	5	Panel	RHABDO
4	*ANO5*	213599.3	c.191dup	Homozygous	5	Panel	BDO
5	*ACADM*	000016.6	c.734C > T	Heterozygous	5	Panel	BD
		000016.6	c.1075G > T	Heterozygous	4		
6	*FKTN*	001079802.2	c.1270G > A	Homozygous	4	Panel	HBDO
7	*PYGM*	005609.4	c.148C > T	Homozygous	5	WES	RHABDO
8	*ACADS*	000017.4	c.1117G > A*	Homozygous	3	Panel	RD
9	*CSNCK2B*	001320.7	c.118A > C	Heterozygous	3	WES	RD
10	*DYSF*	001130987.2	c.3290_3301del*	Heterozygous	3	Panel	RHBD
		001130987.2	c.5347G > C*	Heterozygous	3		
11	*AMPD1*	000036.3	c.133G > T	Heterozygous	3	WES	RD
	*NEB*	001164508.2	c.22642A > G	Heterozygous	3		
	*SPEG*	005876.5	c.2672G > A	Heterozygous	3		
12	*GYG1*	004130.4	c.115G > A	Heterozygous	3	WES	HD
	*POMGNT2*	032806.6	c.1504C > G	Heterozygous	3		
	*POMT2*	013382.7	c.773T > C	Heterozygous	3		
	*TYPM*	001953.5	c.759G > C	Heterozygous	3		
	*KLHL40*	152393.4	c.997C > G	Heterozygous	3		
13	*STIM1*	001382567.1	c.1124T > C	Heterozygous	3	WES	HBD

Abbreviations: A, accustomed physical exercise; ACGM, American College of Medical Genetics and Genomics; B, blood creatine kinase (CK) > 50x ULN; D, drug ingestion/medication/supplements or other exogenous and endogenous factors cannot sufficiently explain the rhabdomyolysis severity; H, hyperCKemia persisting more than 8 weeks after event; O, other family members affected / other exertional symptoms (e.g., cramps, myalgia); R, recurrent episodes of exertional rhabdomyolysis; WES, whole exome sequencing.

Note: * Variants classified as VUS deemed as responsible for the phenotype.

**Table 2:** Clinical data recorded from all patients, divided into two groups based on the underlying genetic cause defined, and their respective *p* -value

Data	Genetic underlying cause defined ( *n* = 9)	Genetic underlying cause not defined ( *n* = 11)	*p* -value
Age at first rhabdomyolysis episode (years)	27 (10–50)	19 (2–33)	0.128**
Sex			
Female	4	8	0.361**
Male	5	3	
Serum CK level at 1st episode (UI/mL)	26.000 (2.235–120.000)	16.100 (6.882–123.000)	0.787**
Serum CK level after at least 8 weeks (UI/mL)	1.198 (123–10.153)	99.5 (64–569)	0.435**
Serum CK ≥ 5x ULN after 8 weeks	5	0	0.0081*
Recurrence of rhabdomyolysis	6	10	0.284*
Exogenous trigger	5	10	0.127*
Fatigue	7	5	0.143*
Exercise intolerance	8	7	0.381*
Myalgia	8	4	0.028*
Cramps	6	3	0.174*
Objective weakness	5	1	0.049*
Family history	5	3	0.361*
Consanguinity	5	0	0.0090*

Notes: *Fisher-Test; **Mann-Whitney U-test.

### Comparison of patients with positive and negative genetic testing

Demographic and clinical data stratified according to the subgroups are shown in Table 2 . There was no statistically significant difference between groups for sex, age at the 1st rhabdomyolysis episode, serum CK peak at first event and serum CK peak after ≥ 8 weeks. All patients matched at least two RHABDO criteria, with the most common reported feature being the ‘D’, which was present for all patients. The second most common feature was ‘R’(recurrence), present in 80% of the sample, although with no statistically significant difference between groups. Indeed, CK ≥ 5x ULN persisting more than 8 weeks after the event was the only feature statistically significant in group comparison, being more prevalent in the group with an underlying genetic cause ( *p* = 0.0081). In the group with a genetic cause determined, there were 5 patients born from consanguineous marriage (parents were cousins of first degree in all cases) and 1 with unknown family history, whereas in the group with negative genetic testing, there was no parental consanguinity reported among 9 patients with known family history ( *p* = 0.0090). There were 2 additional predictors for genetic etiology: presence of myalgia ( *p* = 0.028) and objective weakness ( *p* = 0.049).

## DISCUSSION

The current report is the first published effort addressed to investigate genetic cause for rhabdomyolysis in a Brazilian cohort. We hope to contribute to the genetic background comprehension of this potentially fatal medical condition with remarkable genetic factors of susceptibility in our population. According with previous specialist recommendations, the presence of at least one RHABDO feature should raise suspicion for a genetic cause, 16 but the yield of mnemonic-based genetic investigation in a national sample remained unknown. Interestingly, the outcome of 45% of genetic cause achieved by the two-step work-up employed is comparable to those of international cohorts, reported to be from 11 to 50%. 12 13 Such a variable proportion of positive genetic tests (or also called “positives”) in previous medical reports comes from very heterogeneous cohorts, mainly composed by HyperCKemia, recurrent rhabdomyolysis and metabolic myopathy, as published by Invernizzi et al. 13 Likewise, herein, patients with serum CK ≥ 5x ULN were included, which enabled a higher number of patients to be selected. In contrast, due to limitations of the retrospective data collection, it was not possible to establish a return to baseline in subsequential measures of serum CK in short period as part of the definition of rhabdomyolysis, as performed by Krujit et al., 12 whose study reported a remarkably smaller diagnostic yield (11%). Special emphasis must be placed on it because the current approach probably included some patients who would be better categorized as persistent Hiperckeamia, such as limb-girdle dystrophies that eventually can present with rhabdomyolysis.

Indeed, almost half of the genes identified are related more commonly to limb-girdle dystrophies ( *ANO5* , *DYSF* , *FKTN* ), although pathogenic variants in these genes have already been recognized as factors of genetic susceptibility to the development of rhabdomyolysis. 9 13 16 17 It highlights the fact that structural abnormalities in the sarcoplasm and sarcolemma increase the susceptibility of muscle-cell membrane injury. Therefore, dystrophy-related genes should be considered as potentially causative for rhabdomyolysis in clinical reasoning. On the other hand, the remaining genes ( *PYGM* , related to glycogen storage disease type V—McArdle disease—and *CPT II* , *ACADS* and *ACADM* , all related to fatty-acid metabolism disorders) are well-established causes for recurrent rhabdomyolysis. 7

The variants found in genes *ACADS* (homozygous c.1117G > A) and *DYSF (* c.3290_3301del and c.5347G > C) could only reach VUS classification based on the data available in the medical literature. Despite that, there were pieces of evidence of pathogenicity that could enable us to consider the patients carrying them as part of the group with positive genetic testing. All variants have an extremely low frequency in population database; They are absent in healthy controls and there are no reports of homozygous genotypes in the general population (PM2_M). The genes have a low rate of benign missense variants, and loss of function is a common mechanism of the disease (PP2); Although not specific, the phenotypes are reasonable for both cases with lipid accumulation on muscle biopsy in the patient carrying the homozygous variant in gene *ACADS* (PP4) and a limb-girdle muscle dystrophy pattern with involvement of the posterior compartment of the lower limbs with compatible histopathological findings in the patient carrying the variants in the gene *DYSF* (PP4_S). Besides, multiple computational pieces of evidence support a deleterious effect of the variant found in the *ACADS* gene, such as *Mutation taster* —predicted to be disease causing—, *PolyPhen2* , possibly damaging; Combined Annotation Dependent Depletion (CADD): 27.3, and Rare Exome Variant Ensemble Learner (REVEL) score: 0.7 (PP3). 18 19 20

Furthermore, the results point toward four possible clinical predictors to genetic rhabdomyolysis: persistent CK ≥ 5x ULN, myalgia, objective weakness, and parental consanguinity history. The first two compose the RHABDO criteria and have already been employed in large validation cohorts. It reflects, at least partially, a continuous process of cell-membrane damage with CK release. In contrast, the weakness (defined by any medical council research score different from 5 measured by physical examination performed by a neurologist) suggests an underlying fixed structural damage. It has not been assessed in previous studies and probably was evidenced in the current sample because of the limb-girdle cases encompassed in this cohort. Regardless of the peculiarities of the sample, those four predictors must be taken into account to think of genetic causes for rhabdomyolysis.

It is important to note that the current study has several limitations. First, this is a single center study, which impact the reproducibility of the results around Brazil and other countries. Second, although neuromuscular causes of rhabdomyolysis are rare in the general population, the sample is small. Third, cases reported are from a tertiary neuromuscular disorder clinic, which does not reflect the epidemiological aspects of the general population across the country. Probably, there is selection bias because patients referenced to our clinic often have already been investigated before. This increases the number of unsolved cases in the sample. Besides that, it possibly increases the number of patients with persistent hyperCKemia with underlying dystrophic myopathies included in the rhabdomyolysis cohort. Another bias is the fact that we excluded cases of inflammatory or toxic myopathies. Although this is one of the features that encompass the mnemonic RHABDO, there is some overlap between exogenous and genetic causes. Lastly, there were intrinsic limitations regarding the collection of data, since it is a retrospective analysis.

In conclusion, monogenic causes accounted for nearly half of the cases of rhabdomyolysis without an exogenous cause recognizable in this series. There were multiple genes involved, including some related to ‘classic’ structural myopathies. Based on the predictive factors identified, a genetic testing should be pursued in those patients with persistent CK ≥ 5x ULN after 8 weeks, presence of myalgia, objective weakness, and parental consanguinity history.
